# Current status of experimental models for the study of malaria

**DOI:** 10.1017/S0031182021002134

**Published:** 2022-05

**Authors:** Nelson V. Simwela, Andrew P. Waters

**Affiliations:** Institute of Infection, Immunity & Inflammation, Wellcome Centre for Integrative Parasitology, University of Glasgow, Glasgow, UK

**Keywords:** Animal models, malaria, review

## Abstract

Infection by malaria parasites (*Plasmodium* spp.) remains one of the leading causes of morbidity and mortality, especially in tropical regions of the world. Despite the availability of malaria control tools such as integrated vector management and effective therapeutics, these measures have been continuously undermined by the emergence of vector resistance to insecticides or parasite resistance to frontline antimalarial drugs. Whilst the recent pilot implementation of the RTS,S malaria vaccine is indeed a remarkable feat, highly effective vaccines against malaria remain elusive. The barriers to effective vaccines result from the complexity of both the malaria parasite lifecycle and the parasite as an organism itself with consequent major gaps in our understanding of their biology. Historically and due to the practical and ethical difficulties of working with human malaria infections, research into malaria parasite biology has been extensively facilitated by animal models. Animals have been used to study disease pathogenesis, host immune responses and their (dys)regulation and further disease processes such as transmission. Moreover, animal models remain at the forefront of pre-clinical evaluations of antimalarial drugs (drug efficacy, mode of action, mode of resistance) and vaccines. In this review, we discuss commonly used animal models of malaria, the parasite species used and their advantages and limitations which hinder their extrapolation to actual human disease. We also place into this context the most recent developments such as organoid technologies and humanized mice.

## Graphical abstract

**Figure:**
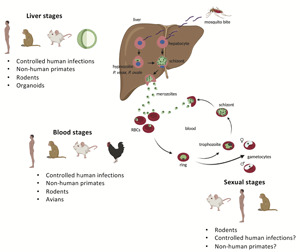

## Introduction

In the annals of public health history, no disease occupies as special a place as malaria. Between 1900 and 2000, up to 300 million people died of the disease accounting for ~5% of all recorded deaths (Carter and Mendis, 2002). Even though significant strides have been made in malaria control over the last 20 years, resource-limited nations in Sub-Saharan Africa (SSA), Amazonia and Southeast Asia remain significantly affected (Feachem *et al*., 2019). In 2019, over 220 million malaria cases were estimated with approximately 400 000 individuals dying of the disease mainly in SSA (>94% for both metrics). Human malaria is mostly caused by *Plasmodium falciparum* and *P. vivax*, the former of which is responsible for the most severe forms of the disease and accounts for the majority of cases in SSA (>99%). Malaria caused by *P. vivax* is also highly prevalent in the WHO South-East Asia and America regions (WHO, 2020). Following earlier sporadic attempts, renewed malaria control programmes have been extensively implemented over the last two decades amidst continued efforts to eradicate the disease. As before, current control strategies mainly revolve around integrated vector management and effective malaria therapeutics as no highly effective vaccine is currently available for the disease. Despite successful implementation of these programmes which have led to significant progress in malaria control efforts over the last 10 years, the emergence of resistance to frontline antimalarial drugs and resistance to insecticides used in vector control are constant and evolving threats. A likely consequence of these threats is that the global incidence of malaria appears to be increasing over the last few years (WHO, 2020).

**Fig. 1.**
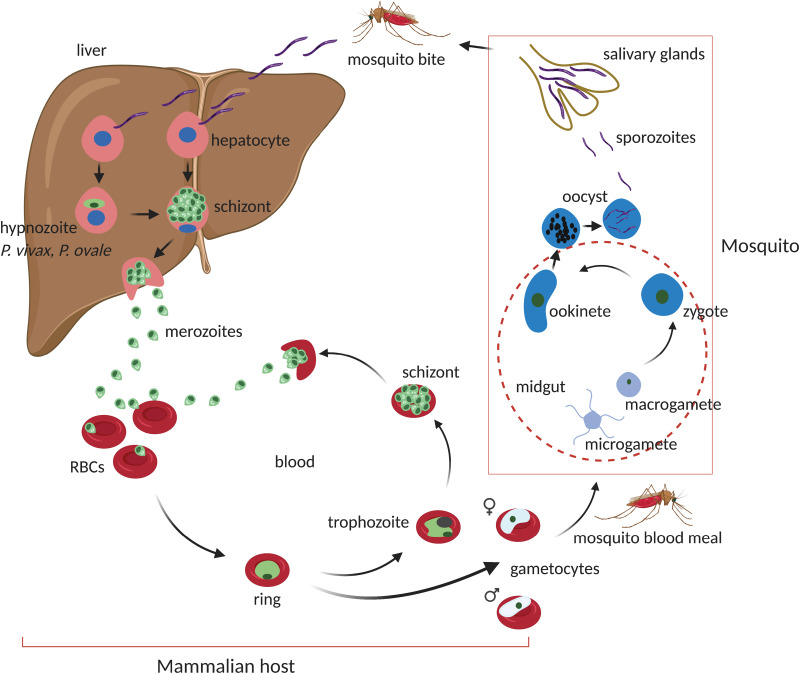
Life cycle of malaria parasite's *Plasmodium* spp. On a mosquito bite, sporozoites are injected at the base of the skin where they migrate through the blood stream and lymph nodes to the liver. In the liver, sporozoites infect hepatocytes to initiate the exoerythrocytic liver stage. Depending on *Plasmodium* spp. (~2 days in *P. berghei* or 6.5 days in *P. falciparum*), sporozoites develop into fully formed mature schizonts containing 29 000–90 000 merozoites after several rounds of asexual proliferation. Upon rupture of the host cell, free merozoites invade red blood cells (RBCs) to initiate the blood-stage cycle. During the liver stage, some sporozoites in certain *Plasmodium* spp. (*P. vivax* and *P. ovale*) can develop into dormant hypnozoite forms which can persist for days to years. The blood stage comprises of a series of asexual developmental transitions; from metabolically less active ring stages to highly active trophozoites which mature to schizonts after another round of asexual proliferation and DNA replication. Mature schizont's carrying species-specific number of merozoites (15–30) rupture to release merozoites which invade new RBCs to re-initiate the cycle. This process usually takes ~48 h in *P. falciparum* and half the time (~24 h) in the rodent malaria *P. berghei*. Meanwhile, during the blood-stage cycle, a small proportion of ring-stage parasites commit to a sexual developmental cycle which results in the formation of male and female gametocytes for transmission. Gametocytes are taken up into a mosquito midgut after a new blood meal where they activate, fertilize and develop into a zygote. The zygote undergoes a meiotic cell division and develops into motile ookinetes which traverse the mosquito midgut to form oocysts. Oocysts go through another round of asexual propagation to generate thousands of sporozoites which migrate to and colonize the mosquito salivary glands to re-initiate the cycle upon a mammalian bite. Figure sketched with BioRender.com.

Fundamental life cycle features of *Plasmodium* spp. are conserved across the genus sharing most of the developmental stages (Fig. 1). Infection of the vertebrate host is initiated by the bite of a female *Anopheles* mosquito which can inject up to 100 sporozoites in experimental conditions. Injected sporozoites move by gliding motility through the extracellular matrix of the skin before eventually invading blood and lymphatic vessels (Ménard *et al*., 2013). Consequently, sporozoites find their way to the liver where they colonize hepatocytes. Through a series of host cell invasions, traversal and exits, sporozoites invade a final hepatocyte where they develop to establish a parasitophorous vacuole (Prudêncio *et al*., 2006; Ménard *et al*., 2013). Thereafter, in an iterative series of DNA replication and asexual proliferation, sporozoites differentiate into mature schizonts which contain tens of thousands of merozoites. In two human-infectious *Plasmodium* species, *P. vivax* and *P. ovale*, the liver stage involves a small proportion of invading sporozoites developing into dormant non-replicating forms called hypnozoites (Krotoski *et al*., 1982*a*; Wells *et al*., 2010). Hypnozoites are characteristically persistent (for up to decades), refractory to most antimalarial drugs and are indeed a frequent source of relapsing malaria caused by *P. vivax.* Mature liver schizonts rupture to release merozoites *via* merosomes which invade red blood cells in the peripheral blood circulation to initiate the asexual blood-stage (ABS) cycle. During this stage, merozoites transit through ring stages to metabolically active trophozoites which massively consumes host haemoglobin and nutrients producing microscopic distinct brown pigments of haemozoin. In mid–late trophozoite stages, the parasite starts replicating its DNA (S-phase) and divides its nucleus to enter the schizont stage. Once mature, schizonts contain, typically, a species-specific 10–30 merozoites, which upon rupture of the infected cell re-invade new red blood cells to initiate another ABS cycle. This results in a cyclic increase in the percentage of infected red blood cells (the ‘parasitaemia’). Schizonts and late trophozoites (of some species) are not usually seen in peripheral circulation as they sequester in the microvasculature (Nishanth and Schlüter, 2019). The ABS cycle of malaria parasites results into most of the disease pathology as the invasion cycles associate with fever episodes and destruction of red blood cells with anaemia and the metabolic consequences of the massive consumption of serum glucose by the parasite. Parasite sequestration in tissues and organs is a hallmark of the most severe forms of the disease especially when sequestration occurs in the brain (cerebral malaria) or placenta (pregnancy-associated malaria) (Miller *et al*., 2002; Storm and Craig, 2014; Nishanth and Schlüter, 2019). During each ABS cycle, a few ring-stage parasites commit to a sexual developmental cycle that produce transmissible forms of the parasite, male and female gametocytes. Commitment is environmentally sensitive and occurs preferentially in young reticulocytes through currently poorly understood mechanisms (Ngotho *et al*., 2019; Venugopal *et al*., 2020). These are taken up by a mosquito during a blood meal and fertilized in the midgut to form a zygote stimulated by mosquito environmental conditions such as low temperature, xanthurenic acid and increased pH (Josling *et al*., 2018; Ngotho *et al*., 2019). A zygote develops into a motile ookinete which traverses the mosquito midgut to become an oocyst. Within the oocyst, thousands of sporozoites develop which are released and infect the mosquito salivary glands ready for transmission to the next mammalian host.

Despite frequent exposure to malaria parasites, ABS long lasting, stage transcending immunity is not known to occur in malaria infections. This has made malaria vaccine development especially challenging despite the recent rollout of the moderately effective but short-term RTS,S vaccine (Draper *et al*., 2018). Attempts to generate ABS malaria vaccines trace back to early seminal work by Cohen and MacGregor in the 1960s, which demonstrated that through passive serum transfer between humans, anti-malarial antibodies could prevent merozoite reinvasion and protect against severe disease (Cohen *et al*., 1961). Such human model approaches to ABS vaccines are now restricted to controlled human infections (consented infections with parasites and or observational sampling of naturally infected individuals), which suffer from their own drawbacks (logistical, ethical and resource requirements). The continued threat to available malaria controls through the emergence and spread of *Plasmodium* parasites that are resistant to current frontline antimalarial drugs as well as vector resistance to commonly used insecticides necessitates the application and use of experimental models that can reproduce (aspects of) human disease while helping in decoding the critical aspects of parasite biology, immunology, and pathogenesis for development of novel interventions. Consequently, animal models have been historically invaluable to the study of malaria. These have ranged from birds, bats, non-human primates (NHPs), rodents and more recently humanized mice and complex three-dimensional *ex vivo* organoids. In this review, we explore different *Plasmodium* spp. which have been used to query human malaria disease in different animal models. With a special focus on NHPs and rodent species, we review the history of human infecting *Plasmodium* that are able to infect rodents and NHPs. We also outline the history of rodent-specific *Plasmodium* in the wider context of disease similarity with human malaria. More importantly, the review discusses the aspects of parasite biology and malaria disease processes that these animal models are helping to unravel. We also explore some recent advancements in controlled human infections with human *P. falciparum* and the consequent implications on future use of different animal models in malaria research.

## Human infectious *Plasmodium* in NHPs and immuno-compromised mice

Over 100 *Plasmodium* spp. are known to exist, of which only five (*P. falciparum*, *P. malariae*, *P. vivax, P. knowlesi* and *P. ovale*) cause disease in humans. Despite its discovery as the main causative agent of malaria in humans in 1880 by the French Army physician Charles Laveran, *P. falciparum* did not inherit its widely used name (often called by different names; *Ematozoo falciforme, Haematozoon falciforme, Haematozoon falciparum*) until its certification by the International Commission on Zoological Nomenclature over 70 years later in 1954 following years of debate on the naming system (Bruce-Chwatt, 1987). *P. falciparum* remains the main cause of the most severe and lethal forms of human malaria. *P. falciparum* studies have been, almost, entirely been restricted to *in vitro* culture systems mostly due to the exquisite host specificity as only humans and a very few NHPs are susceptible to infection (Schuster, 2002). Since a stable *in vitro* culture system was established and reported for *P. falciparum* in 1976 by Trager and Jensen (Trager and Jensen, 1976), similar culture systems have been adapted (to a certain extent) for all the five human infectious *Plasmodium* (HIP) (Schuster, 2002). These *in vitro* systems have indeed been the backbone into the study of human malaria parasite biology and antimalarial drug discovery programmes for the last 40 years. However, despite the availability of a wide number of *P. falciparum* field and culture adapted isolates, application of *in vitro* systems cannot entirely replicate the *in vivo* environment particularly with regard to host genetic heterogeneities, the physicality of development under conditions of blood flow and sequestration as well as immune pressure (LeRoux *et al*., 2009). Furthermore, due to the absence of selection pressure to transmit, it is common for lab isolates to lose the ability to produce gametocytes *in vitro* after prolonged cultivation. The use of animal models in these cases, has thus, often, been indispensable. The first strain of *P. falciparum* that was able to infect New World NHPs was adapted from a Ugandan isolate and reported in 1967 (Geiman and Meagher, 1967). Since then, several *P. falciparum* isolates (FCH/4, Indochina I, Geneve, Salvador I, Panama II etc) that infect splenectomized or non-splenectomized monkeys from the American tropics have been reported and are archived by the US Centre for Disease Control (Galinski and Barnwell, 2012). These isolates have been widely used to study human malaria disease in NHP (see sections below). However, due to the high costs required to maintain NHP under laboratory conditions, lack of animals and ethical problems, the use of *P. falciparum* strains in NHP is very limited. Over the years, *P. falciparum* studies *in vivo* have been revitalized by the development of humanized mice and experimental human challenge models. Humanized mice (mice that express human genes or have been engrafted with human tissues) are becoming attractive and suitable models as a potential substitute to NHPs. They are generated by transplantation with either primary human hepatocytes or red blood cells under immunodeficiency conditions to prevent xenorejection. Because various organs and or tissues can be transplanted into mice, this is becoming a more convenient approach to modelling several aspects of malaria parasite biology as well as disease pathogenesis. However, humanized mice still lack sufficient tissue or organ penetration of the engrafted cells due to the hostile mouse microenvironment for foreign human cells to survive and proliferate. Moreover, the use of immunocompromised mice limits the study of certain aspects of host–pathogen interactions such as host immune responses. Nevertheless, as the development of these humanized mice continue to improve, their utility and full potential will hopefully be realized, reviewed in detail by Kaushansky *et al*. (2014), Siu and Ploss (2015) and Minkah *et al*. (2018).

## NHPs and rodent infecting *Plasmodium*

### Rodent malaria

**Fig. 2.**
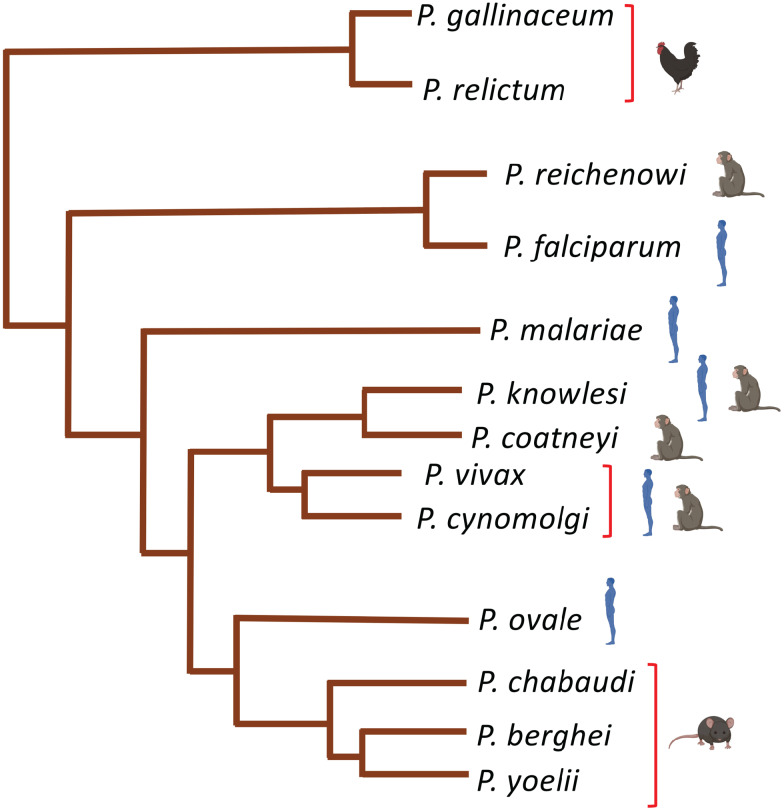
Phylogenetic relatedness of *Plasmodium*'s spp. The tree is based on recent published genomes of the indicated *Plasmodiu*m spp. and was adapted with minor modifications from Rutledge *et al*. (2017), Böhme *et al*. (2018).

Malaria parasites that infect rodents (mice and rats) have been extensively used to study and model human disease *in vivo*. Four species of rodent malaria parasites (RMPs i.e. *P. berghei, P. yoelii, P. chabaudi, P. vinckei*) that were originally isolated on various occasions in Central African thicket rats have been at the centre of these studies. These parasite species share a highly conserved chromosomal gene synteny with the human infecting *P. falciparum* (Carlton *et al*., 1998*b*) with, however, subtle differences in stage-specific morphologies, duration of life cycle and host cell preferences (Table 1). *P. chabaudi* and *P. vinckei* have the ability to invade mature red blood cells and achieve high parasitaemia just like human *P. falciparum* and *P. malariae*, whilst *P. berghei* and *P. yoelii* are generally restricted to reticulocyte invasion which is similar to human infecting *P. vivax* and *P. ovale* (Table 1). Nevertheless, the basic biology of rodent- and human-infectious *Plasmodium* is fundamentally conserved. This has allowed for their use in studying several aspects of malaria parasite development, host–pathogen interactions, drug efficacy evaluations and vaccine studies which would otherwise be inaccessible with *P. falciparum in vitro*. Furthermore, RMPs offer a plethora of advantages among which include their ease of handling in rodents, experimental tractability of all life cycle stages under lab conditions as well as the availability of a wide array of genetic manipulation systems. Still, RMPs and their hosts are both divergent from their human equivalents (*P. falciparum* and humans) (Fig. 2). Even though they can provide crucial insights into the conserved elements of parasite biology, the finer molecular details might be different from their human counterparts due to the unique aspects of their hosts. The use of these models should therefore be pertinently tailored to the biological question under study through direct comparison to human parasites (Craig *et al*., 2012; De Niz and Heussler, 2018).

**Table 1.** Comparison of human, rodent and primate *Plasmodium*'s in relation to key life cycle features, host tropisms and disease pathogenesis

	Human *Plasmodium*				Rodent *Plasmodium*				Primate *Plasmodium*		
*Plasmodium* spp.	*P. falciparum*	*P. vivax*	*P. malariae*	*P. ovale*	*P. berghei*	*P. yoelii*	*P. chabaudi*	*P. vinckei*	*P. cynomolgi*	*P. coatneyi*	*P. knowlesi*
Commonly used strains	3D7, NF54, Dd2, 7G8, HB, FCB	Sal-1	PmUG01	PocGH01, PowCR01	NK65, ANKA, K173	17X, YM	AS	ATCC 30091	M, B, Berok	Hackeri	A1, H
Blood stage duration	~48 h	48 h	72 h	50 h	22–24 h	18 h	24 h	24 h	~48 h	~48 h	~27 h
Liver stage duration	~144–168 h	~192 h	~360 h	120–216 h	50 h	50 h	52–53 h	53–61 h	~192 h	156–192 h	~120 h
Gametocytogenesis, duration and morphology	10–12 days, five morphological stages (Fig. 1)	2 days, spherical	~3 days spherical	~2 days spherical	1 day, spherical	1 day, spherical	~2 days spherical	~1.5 days spherical	~3–4 days spherical	~2 days spherical	~2 days spherical
Host cell tropism	Mature RBCs	Reticulocytes	Mature RBCs	Reticulocytes	Reticulocytes	Reticulocytes	Mature RBCs	Mature RBCs	Reticulocytes	Mature RBCs	Mature RBCs
Synchronicity of blood-stage life cycle	Yes	No	Yes	Yes	No	No	Yes	Yes	No	Yes	Yes
Virulence ligands	PfEMP1	PvDBP	Unknown	Unknown	Unknown	YIR	PIR	Unknown	DBP	SICA	SICA
Tissue sequestration	Multi-organ; spleen, brain, bone marrow, liver, and lungs	Multi-organ; spleen, bone marrow, liver, and lungs	Yes; specific organs not characterized	Yes; specific organs not characterized	Multi-organ; mostly in the liver, spleen and lungs	Yes; specific organs not characterized	Yes; mostly in the liver	Yes; specific organs not characterized	Yes; specific organs not characterized	Multi-organ; spleen, brain, liver, heart, and lungs	Multi-organ; mostly in the brain, heart and kidneys
Natural host	Human	*Aotus lemurinus lemurinus*	Human	Human	*Grammomys surdaster*	*Grammomys surdaster*	*Grammomys surdaster*	*Grammomys surdaster*	Macaques	Macaques	Macaques

### NHPs malaria

For decades, NHPs have also proved invaluable to studying malaria disease pathogenesis as well as fundamental aspects of parasite biology, reviewed by Galinski and Barnwell (2012). Crucially, *P. cynomolgi* and *P. vivax* hypnozoites (dormant liver stages) were first discovered in NHPs, specifically in rhesus macaques for the former (Krotoski *et al*., 1982*b*) and in Chimpanzees for the latter (Krotoski *et al*., 1982*a*). Due to a reticulocyte invasion dependency (Gruszczyk *et al*., 2018; Malleret *et al*., 2021) (Table 1), *P. vivax* lacks a robust *in vitro* culture system unlike *P. falciparum*. NHPs have thus been instrumental for understanding the biology of this parasite. Over 40 *P. vivax* strains that are able to infect NHPs, specifically New World monkeys of the *Aotus* and *Saimiri* species have been archived by the CDC (Galinski and Barnwell, 2012). By infecting splenectomized NHPs (to minimize splenic clearance of infected red blood cells) through mosquito bites or intravenous injection of purified sporozoites, chronic relapsing *P. vivax* malaria can be reproduced in these models (Joyner *et al*., 2015). However, due to difficulties in isolating enough sporozoites from *P. vivax*, a closely related sister species, *P. cynomolgi* (Fig. 2) is often used as a model for *P. vivax* in NHPs as this parasite can be used to generate millions of sporozoites within a short period of time (Rosenberg and Rungsiwongse, 1991; Joyner *et al*., 2015). *P. cynomolgi* and *P. vivax* share a highly conserved genome organization (GC content ~41%, equal number of positionally conserved centromeres etc) (Tachibana *et al*., 2012) as well as similar features of disease pathology such as the formation of hypnozoites. Unlike *P. vivax* which is mostly restricted to New World monkeys, *P. cynomolgi* strains that infect larger Old World monkeys (macaques) have been successfully adapted providing, in the absence of long-term *in vitro* culture, the only means to generate sufficient amounts of parasite material for downstream analyses (Joyner *et al*., 2015). Moreover, *P. cynomolgi* is easily amenable to genetic manipulation systems allowing for interrogation of gene function systems which have largely been inaccessible in *P. vivax* (Voorberg-van der Wel *et al*., 2013; Joyner *et al*., 2015). *P. cynomolgi* remains the main model of understanding *P. vivax* biology among which include evaluating the efficacy of antimalarial drugs with potential activity in difficult to eliminate hypnozoite stages, reviewed by Zeeman and Kocken (2017).

Other NHP infecting malaria parasites include *P. coatneyi* and *P. knowlesi*. *P. coatneyi* was first isolated in the early 1960s from a forest mosquito *Anopheles hackeri* in Malaysia (Eyles *et al*., 1962). Only one isolate of *P. coatneyi* (the Hackeri strain) has been characterized and preserved by the CDC ever since (Galinski and Barnwell, 2012). In macaques, *P. coatneyi* infection is highly similar to *P. falciparum*, sharing almost the same aspects of parasite morphology and disease pathogenesis. Late stages of *P. coatneyi* (trophozoites and schizonts) are known to sequester in tissues and organs just like human *P. falciparum*. *P. coatneyi* infected red blood cells also exhibit rosetting characteristics as is commonly seen in *P. falciparum* (Udomsangpetch *et al*., 1991). Crucially, most features of severe malaria pathogenesis; high parasitaemia, anaemia, parasite tissue sequestration, coma and cerebral malaria are all known to occur in *P. coatneyi* infected NHPs. This has made this species of *Plasmodium* a suitable model for a comparative analysis of human malaria disease pathogenesis despite a lack of close intrinsic genetic similarity (Mitsui *et al*., 2010) (Fig. 2). A zoonotic *Plasmodium*, *P. knowlesi* is another NHP infecting malaria parasite which was first isolated in the 1930s (H, Nuri, Hackeri strains) in macaques (Galinski and Barnwell, 2012). *P. knowlesi* is the only NHP malaria parasite with a relatively short life cycle (24 h) that often results in a rapid increase in parasitaemia and death of the host in the absence of treatment. Even though this parasite can infect humans, in what has been some of the best-described cases of zoonotic malaria (Singh and Daneshvar, 2013), differences in the duration of life cycle and disease presentation has restricted the utility of *P. knowlesi* as a model of *P. falciparum*. Nevertheless, *P. knowlesi* infections in macaques have been used to study host immunity to malaria, merozoite invasion biology as well as antigenic variation mechanisms (Galinski and Barnwell, 2012; Singh and Daneshvar, 2013). In fact, the first merozoite invasion receptor ligands on the surface of host red blood cells, Duffy group antigens, were identified using this species of *Plasmodium* (Miller *et al*., 1975).

### Other *Plasmodium* spp. in animals

Before the discovery of RMPs (*P. berghei* in 1949), avian malaria parasites were the experimental spp. of choice for studying malaria parasite biology as they were discovered at almost the same time as *P. falciparum* (Huff and Bloom, 1935; Raffaele and World Health, 1965; Pigeault *et al*., 2015*b*). Avian malaria parasites comprise of an unknown number of species (>55) belonging to two genera, *Plasmodium* and *Haemoproteus* (Atkinson *et al*., 2000; Valkiūnas and Iezhova, 2018). Meanwhile, avian malaria is mostly caused by *P. relictum* (subgenera *Haemamoeba*) which is endemic to all parts of the world except Antarctica (Bensch *et al*., 2009). Other causative agents include *P. gallinaceum* and *P. lophurae* which occur at less frequency and can be a significant problem in poultry industries (Coggeshall, 1938; Springer, 1991). Despite conserved life cycle features with other *Plasmodium*, avian malaria parasites have a slightly different life cycle. They display a low host specificity across bird species as well as a marked variation in developmental patterns in various hosts (Bensch *et al*., 2009; Hellgren *et al*., 2015). Avian malaria parasites also appear to produce dormant parasite forms both in the liver and ABS cycles as opposed to other *Plasmodium* such as *P. vivax* which produce the same only during the liver stage (Cosgrove, 2005). Moreover, avian red blood cells are nucleated. This provides steady access to nutrients and metabolite transport mechanisms (through the host) which would obviously contribute to divergent evolutionary trajectories of these parasites as compared to their mammalian counterparts (Böhme *et al*., 2018). Broadly, avian malaria is usually sublethal (in endemic areas) while in some cases it can lead to severe disease especially in cases of accidental introduction in non-endemic areas (Atkinson *et al*., 2000). After the discovery of RMPs, experimental malaria almost, entirely, switched to rodents (Rivero and Gandon, 2018). However, in recent years, avian malaria parasites have re-emerged as appropriate models to studying malaria parasite ecology and evolution mostly due to their rich genetic and phenotypic diversities (Pigeault *et al*., 2015*a*, 2015*b*; Rivero and Gandon, 2018). Complete genomes for *P. relictum* and *P. gallinaceum* have now been published (Böhme *et al*., 2018). Avian malaria parasites have also played some vital historical role not just in understanding parasite ecology and evolution but also technology development. In fact, the first genetic transformation of a malaria parasite was achieved in *P. gallinaceum* (Goonewardene *et al*., 1993).

## Applications of animal models in malaria

### Pathogenesis of severe malaria

In humans, malaria disease spectrum varies significantly between children and adults. These differences can range from no symptoms at all in asymptomatic individuals, mild disease in some and in a few cases (<2%) severe and lethal forms of the disease. Severe malaria is often characterized by a multi-organ system involvement presenting as anaemia, metabolic acidosis or cerebral malaria (CM) (Miller *et al*., 2002). CM remains the leading cause of malaria-related deaths in children under the age of five in SSA (Ghazanfari *et al*., 2018). Irrespective of successful antimalarial treatment, >15% mortality rates occur in CM patients (Newton and Krishna, 1998). In areas of low malaria transmission like SEA, CM does not just occur in children as the disease is also common in adults (Ghazanfari *et al*., 2018). Despite intensive research efforts, the pathogenesis of CM remains poorly understood. Studies using post-mortem samples have, however, pointed to tissue and organ sequestration of parasite-infected red blood cells as a pathological hallmark of all human CM cases (Berendt *et al*., 1994; Taylor *et al*., 2004). Sequestration of infected red blood cells to the vascular endothelium of tissues is mediated by a parasite ligand expressed on the surface of red blood cells, the polymorphic *P. falciparum* erythrocyte membrane protein-1 (PfEMP-1) that undergoes antigenic variation (Jensen *et al*., 2020). Several host endothelial receptors bound by different forms of PfEMP-1 have been identified among which include CD36, the intercellular adhesion molecule 1 (ICAM-1) and the endothelial protein C receptor (EPCR) (Berendt *et al*., 1989; Gamain *et al*., 2002; Brown *et al*., 2013; Avril *et al*., 2016). It has been proposed that parasite sequestration through PfEMP-1 engagement of host receptors causes a mechanical occlusion of blood vessels which reduces blood flow and delivery of oxygen and nutrients into various tissues and organs. In CM, this has been associated with hypoxia, coma and death, the classical clinical features of the disease (Taylor *et al*., 2004). However, besides parasite sequestration, immune effector cells and platelet accumulation also appear to be important drivers of CM pathogenesis (Grau *et al*., 2003; Hochman *et al*., 2015). Engagement of human endothelial receptors by infected red blood cells is also known to trigger local signalling processes through angiogenic factors angiopoietin-2 (Ang-2) and vascular endothelial growth factor (VEGF) both of which contribute to or are important drivers of CM pathogenesis (Yeo *et al*., 2008). Thus far, a well-characterized model of human CM remains the *P. berghei* ANKA strain infection of several mouse strains, specifically the C57BL/6 mouse lineages. In this experimental CM (ECM) model, mice develop similar neurological features to human CM which among others include paralysis, coma and death (Hunt *et al*., 2010*a*). However, some parasitological features of human CM are not apparent in ECM. For instance, sequestration of infected red blood cells does not appear to occur in the *P. berghei* ECM model. In some observations, parasite sequestration in *P. berghei* ANKA occurs in the lungs and involves host receptors such CD36 but accumulation in the brain does not correlate with disease pathogenesis (Hearn *et al*., 2000; Franke-Fayard *et al*., 2005). This in contrast to other studies which have shown that accumulation of parasitized red blood cells in the brain is necessary for the onset of ECM-related pathology (Amante *et al*., 2010; Baptista *et al*., 2010; Claser *et al*., 2011). More recently, quantitative brain mapping of mice with ECM but infected with a different *P. berghei* strain (NK65) revealed that parasite-infected red blood cells occluded brain micro-vessels, a feature which was not observed in similar mouse infections with mild disease (Strangward *et al*., 2017). In human CM, death is thought to be primarily due to brain swelling depressing the fundus which regulates breathing (Seydel *et al*., 2015), a phenomenon which is yet to be demonstrated in ECM. Even though an increase in brain volume could be the result of immune cell infiltration and parasite sequestration, both of which are also common in ECM, care should be taken in the use of the ECM model to the study of human CM (Craig *et al*., 2012).

*P. berghei* ECM also appears to be largely an inflammatory syndrome characterized by immune cell infiltration and accumulation of pro-inflammatory cytokines such as IFN-*λ* and lymphotoxin *α* (Grau *et al*., 1989*a*, 1993; Engwerda *et al*., 2002; Ghazanfari *et al*., 2018). This is relatively divergent from human CM, as despite signs of vascular damage and inflammation in some cases of human CM (Grau *et al*., 1989*b*; Kwiatkowski *et al*., 1990; Taylor *et al*., 2004), these have been largely refuted as proinflammatory markers appear to poorly associate with human CM in the brains of diseased children (Conroy *et al*., 2010; Erdman *et al*., 2011). The benefits of the *P. berghei* ECM model are also challenged by potential intervention approaches that can emerge in such experimental systems. Divergent pathologies between ECM and human CM, be it in cytoadherence biology or immune-based infiltration mechanisms question the utility of the ECM model in evaluating potential inhibitor or adjuvant therapies that are intended for eventual use to treat human CM. Indeed, such interventions, which have relied on or were based on immune mechanisms observed in ECM and to a limited extent, pathological features in some human cases have largely been unsuccessful (Kwiatkowski *et al*., 1993; Prasad and Garner, 2000; Lell *et al*., 2010). Moreover, there appears to be a divergent spectrum in *P. berghei* ECM phenotypes in different labs, animal strains and *P. berghei* isolates (Amani *et al*., 1998). Standardization of protocols and readouts from such models would thus help in interrogating the important features of ECM and associated interventions and their extrapolation to human CM. More recently, alternatives to the *P. berghei* ECM model have seen the development and application of humanized SCID mice, into which, human *P. falciparum* cytoadherence and human CM can be directly modelled. Several humanized mice that can recapitulate human liver and blood-stage malaria disease have been reported, reviewed by Minkah *et al*. (2018). Some early work using some of these mice (engrafted with human red blood cells) has however, demonstrated that *P. falciparum* infections in these models do not cytoadhere nor lead to CM (Angulo-Barturen *et al*., 2008). Nevertheless, more recent work, using similar models, in which human vasculature was implanted and allowed to anastomose locally into the host circulatory system has demonstrated a level of *in vivo P. falciparum* cytoadhesion that will hopefully be useful and refined further to model this aspect of human CM (Meehan *et al*., 2020) leading to potential interventions.

Due to their close relatedness to humans, NHPs have often been promoted as potential powerful alternatives to modelling human CM. NHPs infections with *P. coatneyi*, a simian malaria parasite, phenotypically resemble the vital aspects of *P. falciparum* induced human CM such as parasite rosetting, sequestration and the associated pathological features (Udomsangpetch *et al*., 1991; Kawai *et al*., 1995). Similarly, infections of macaques with *P. knowlesi* results in severe disease that is lethal to the host within days with a partial sequestration of infected red blood cells in various tissues (Singh and Daneshvar, 2013). Unlike *P. berghei* which lacks the var gene repertoires that encode for PfEMP-1 in *P. falciparum*, both *P. coatneyi* and *P. knowlesi* possess var like multigene families (SICAvar) which seemingly mediate parasite adhesion properties and antigenic variations (al-Khedery *et al*., 1999; Chien *et al*., 2016). Thus, NHPs would offer better models of human CM as better tools to study the neurological aspects of CM such as advanced imaging technologies like MRIs which are revealing important aspects of this disease in humans (Seydel *et al*., 2015) could easily be applied to NHPs. However, this is unlikely as research in these models has been limited by lack of investment and the growing campaigns in the scientific community to entirely abandon the use of NHPs in biomedical research.

### Liver stage biology, blood-stage immunity and vaccine development

#### Animal models of malaria liver stages

Successful transmission of malaria parasites to mosquito vectors (Fig. 1) require inoculation of sporozoites into a mammalian host to initiate a pre-erythrocytic stage in the liver. Unlike blood stages, liver-stage malaria parasites are usually asymptomatic and are often very difficult to produce *in vitro*. Furthermore, there are the obvious ethical infeasibilities of using the human liver to study such stages *in vivo*. Thus, RMPs, *P. berghei* and *P. yoelii,* have traditionally been used to probe malaria liver stage biology (Ménard *et al*., 2013). They have been extensively utilized, not just to study liver disease progression, but also the resulting host immunity. Indeed, some of the early seminal work demonstrated that vaccine challenge of mice with irradiated *P. berghei* sporozoites induced protective immunity to subsequent parasite challenges (Nussenzweig *et al*., 1967). These early studies have been successfully progressed with the current ability to general sporozoite-based vaccine candidates through knockout of specific genes that are conserved in human-infectious *Plasmodium* or live inoculation with infectious parasites. These approaches elicit protective immune responses by exposing the host to antigens from parasites with arrested development in the liver or through treatment of blood-stage infection upon live sporozoite challenges, reviewed by Goh *et al*. (2019). Further to that, both of these parasites have been used to demonstrate the role of humoral and T-cell immunological responses to liver stages of malaria parasites. In *P. yoelii*, earlier work on monoclonal antibodies raised against a circumsporozoite (CSP) protein were shown to provide immune protection to mice by blocking sporozoite-mediated invasion of hepatocytes (Charoenvit *et al*., 1991). Recently, it has been further demonstrated that immunization of mice with late-stage arresting sporozoites that lack a type II fatty acid synthesis enzyme that has been genetically ablated (*Pyfabb/f−*) result in robust humoral as well as CD4 T-cells immune responses capable of blocking follow-up challenges with wild-type sporozoites (Vaughan *et al*., 2009; Butler *et al*., 2011; Keitany *et al*., 2014). Similarly, vaccine challenges with a monoclonal antibody (IG1, k) against CSP have been shown to confer protection to mice exposed to otherwise lethal inocula of *P. berghei* parasites (Potocnjak *et al*., 1980). Despite the remarkable utility of these rodent RMPs in studying liver stages, significant differences and evolutionary divergences between RMPs and HIPs (Fig. 2) as well as their hosts means biological implications from such models must be very carefully extrapolated. For instance, *P. berghei* sporozoites can transform into infectious merozoites completing the extraerythrocytic cycle in skin fibroblasts at the site of inoculation of mice without requiring a hepatic cycle (Gueirard *et al*., 2010). This phenomenon also occurs in *P. yoelii* but no blood-stage infection ensues (Voza *et al*., 2012). Moreover, differences in duration of liver-stage cycle between human and RMPs (Table 1) as well as the inability of *P. berghei* and *P. yoelli* to form hypnozoites (persistent liver stages) further alludes to the complications of using such models to broadly inform on the liver stage biology of all HIPs. Thus far, *P. cynomolgi* remains the only robust NHP malaria parasite that is extensively used to study *P. vivax* liver stage biology due to their close relatedness (Fig. 2) (Pasini *et al*., 2017) and experimental tractability (Zeeman and Kocken, 2017). The recent adaptation of *P. cynomolgi* to a robust *in vitro* culture system (Chua *et al*., 2019*b*) and the development of human and simian derived hepatocyte organoids which can support *ex-vivo* propagation of both *P. cynomolgi* and *P. vivax* (Chua *et al*., 2019*a*) will hopefully help in understanding fundamental aspects of these parasites biology (Table 2).

**Table 2.** Common applications of animal models of malaria

Model	*Plasmodium* spp.	Disease form	Common applications
Rodents (mice and rats)	*P. berghei*	Induces severe disease (anaemia, cerebral malaria). Disease pathology varies between *P. berghei* strains and mouse lineages	ECM, *in vivo* drug efficacy evaluation, model for other forms of severe malaria (anaemia, placental malaria)
	*P. yoelii*	Can induce severe disease (anaemia, cerebral malaria). Some strains are non-lethal (17XNL) while others are fatal (17XL). They are significant differences in disease pathology in the lethal 17X and YM strains	Primary model for host–parasite receptor–ligand engagement, immune mechanisms to blood-stage stage disease and vaccine development, pathogenesis and ECM.
	*P. chabaudi*	Usually non-fatal and results in a chronic infection	Immune mechanisms, drug resistance and efficacy model, antigenic variations, general disease pathogenesis
	*P. vinckei*	Seemingly fatal although most disease pathology states have not been studied extensively	Immune mechanisms, drug resistance and efficacy model, general disease pathogenesis
Humanized mice	*P. falciparum*	Standard malaria disease pathology (non-severe)	Pre-erythrocytic biology of malaria parasites, vaccine development for blood-stage infection
Non-human primates	*P. falciparum* (GB4, FVO, FUP strains)	Standard malaria disease pathology severe anaemia	Malaria vaccine efficacy evaluations, model of malaria-induced human anaemia, drug efficacy evaluations
	*P. knowlesi*	Standard malaria disease pathology, extremely fatal when left untreated	Virulence studies, malaria immunity, antigenic variation, merozoite invasion biology and other malaria pathologies
	*P. coatneyi*	Similar malaria disease pathology as *P. falciparum*	An important model for studying the pathophysiology of severe malaria
	*P. cynomolgi*	Mild malaria disease that resembles human infecting *P. vivax*	Most reliable model for studying human malaria caused by *P. vivax*

In the face of some of the above caveats, human liver-stage biology has been continuously studied using functional *in vitro* assays. These have included exploratory evaluation of inhibitory activities of immune sera raised against sporozoites infecting cultured human hepatocytes (Huhep) (Kaushansky *et al*., 2012; Seder *et al*., 2013). However, the lack of robustness in these assays and the absence of the complex three-dimensional architecture of the ideal liver microenvironment always limit their physiological relevance. The recent development of humanized mice has provided promising models which can indeed capture human liver-stage biology, to extents, which have thus far been unexplorable (Minkah *et al*., 2018). Human liver chimaeric mice are currently available to examine the biology of sporozoite invasion of hepatocytes (Vaughan *et al*., 2012; Mikolajczak *et al*., 2015). In these mice, huHep are engrafted under severe combined immunodeficiency (SCID) followed by depletion of host hepatocytes to allow for propagation of huHep in mice liver parenchyma. huHep mice models on SCID background have demonstrated successful infection with *P. falciparum* sporozoites that develop into liver-stage schizonts which in turn release exo-erythrocytic merozoites (Morosan *et al*., 2006; Sacci *et al*., 2006). These humanized huHep mice have also been used to explore the role of human immune responses to liver stages of malaria parasites. Passive immunization of huHep mice with monoclonal antibodies raised against *P. falciparum* sporozoites has been shown to block the establishment of liver-stage infection (Foquet *et al*., 2014). Mice transplanted with human immune system components (CD4, CD8 or B cells) have also been developed and used to characterize equivalent human immune responses to liver stages of malaria parasites. These mice were produced in immunocompromised backgrounds by adenoviral transduction of human HLA class II alleles followed by engraftment with haematopoietic CD34+ cells, which can proliferate to different immune cell subsets. Immunization of these mice with *P. falciparum* CSP provided protection to transgenic *P. berghei* infections carrying *P. falciparum* CSP (Huang *et al*., 2015). Nevertheless, the current generation of humanized mice lack adequate tissue penetration of engrafted cells while the requirement for the use of SCID conditions to maintain such cells in the host limits their use in probing certain aspects of parasite biology. Whilst they remain highly artificial, the evolution and further refinement of these models is, however, opening new exciting avenues into not just the *in vivo* liver stage biology of malaria parasites, but other stages as well (Minkah *et al*., 2018).

#### Animal models of malaria blood stages and vaccine development

Besides their utility in the study of severe disease (discussed above), malaria animal models have also been instrumental in understanding host immune responses and the biology of erythrocytic blood stages. Particularly, RMPs together with longitudinal studies of experimental human infection challenges (Sauerwein *et al*., 2011) have been extensively used to identify host immune responses that control blood stages of malaria parasites. In the *P. chabaudi* blood-stage infection model (the most widely used model for these stages), IgG antibody responses, T helper 1 cells and proinflammatory cytokines (Interleukin 12 and interferon *λ*) have been shown to control the onset of acute infection and progression to peak parasitaemia (Meding and Langhorne, 1991; Su and Stevenson, 2002). *P. chabaudi* and *P. berghei* infections have also been used to establish the mechanisms of parasite and host-mediated immune evasion through dysregulation of B-cell humoral responses (Ryg-Cornejo *et al*., 2016). Despite inducing the proliferation of T-helper cells, *P. berghei* and *P. chabaudi* infections in mice also appear to block further differentiation of these cells which in turn inactivates downstream induction of protective B cell responses (Ryg-Cornejo *et al*., 2016). Similar levels of infection-induced immune dysregulations have been observed in *P. falciparum* infections of humans in experimental challenge approaches illustrating host response convergences which can be exploited to develop suitable interventions (Montes de Oca *et al*., 2016). Liver chimaeric mice which can sustain a low-level parasitaemia in the blood if transfused with human red blood cells are also an emerging model of human ABS (Mikolajczak *et al*., 2015). However, parasites are only maintained for a short period of time due to the rapid clearance of the infused human red blood cells. Further manipulations of the host immune system coupled to daily injections of fresh human red blood cells in these mice is proving promising as parasites reproduce features of human disease pathology such as tissue sequestration. The use of such mice in evaluating the efficacy of antimalarial drugs and the suitability of vaccine antigen targets is rapidly becoming an option (Foquet *et al*., 2018). These approaches are, however, inefficient to reproduce at scale. The development of mice with the capability to produce human red blood cells through engrafting of haematopoietic stem cells in the bone marrow would hopefully provide better and more robust models especially for reticulocyte tropic parasites such as *P. vivax*.

As discussed above, RMPs (specifically *P. berghei, P.chabaudi* and *P. yoelii*) have been remarkably useful in dissecting the liver and blood-stage biology of malaria parasites. This is in addition to host immune responses to these stages which are the basis of current malaria vaccine developments through either the attenuated liver and ABSs or their subunits (Draper *et al*., 2018). Successful experimental reciprocation in humans has also been achieved as up to 100% protection is possible in humans vaccinated with irradiated *P. falciparum* sporozoites under experimental challenge infections (Hoffman *et al*., 2015). Other malaria vaccine strategies which were pioneered in mice include vaccinations with fully infectious sporozoites administered with chloroquine prophylaxis (to block blood-stage disease) and the use of genetically attenuated parasites (GAPs) that lack components required for progression through the liver (Beaudoin *et al*., 1977; Mueller *et al*., 2005). In human *P. falciparum* vaccine and infection challenges, inoculation with live sporozoites covered by chloroquine prophylaxis has proved to be moderately efficacious (~66% efficacy rates) (Roestenberg *et al*., 2011). However, human clinical trials with GAP-based *P. falciparum* vaccines resulted in the establishment of blood-stage infection despite robust immune responses illustrating the limitations of directly extrapolating some preclinical findings in RMPs to humans due to host genetic diversities (Spring *et al*., 2013). Similar discrepancies have been observed with subunit-based vaccines such as the CSP DNA vaccine which elicited potent immune responses in *P. berghei* and *P. yoelli* (Hoffman *et al*., 1994; Sedegah *et al*., 1994) but did not confer any protection in humans (Richie *et al*., 2012). In these situations, since it only requires a single successful merozoite invasion to initiate a blood-stage cycle, liver-stage vaccines would (theoretically) need to be 100% efficacious to prevent disease which, in part, could explain some of the divergent vaccine responses between RMPs and humans. Similarly, apical membrane antigen (AMA-1)-based vaccines targeting blood stages which demonstrated efficacy in *P. knowlesi* (*rhesus* macaques) and *P. chabaudi* (Anders *et al*., 1998; Mahdi Abdel Hamid *et al*., 2011) resulted in limited to no protection with *P. falciparum* equivalents in humans (Thera *et al*., 2011). Still, RMPs and controlled human infection models are still providing complimentary toolsets that assist in the production of better and more potent vaccines (Minkah *et al*., 2018; Stanisic *et al*., 2018). NHPs have also been used in pre-clinical malaria vaccine evaluations. Inoculation of *rhesus* monkeys with irradiated *P. knowlesi* sporozoites elicited CD8+ T cell immune response that were strongly protective to subsequent parasite infections (Weiss and Jiang, 2012). *Aotus* monkeys inoculated with a recently identified *P. falciparum* reticulocyte-binding protein homologue 5 (PfRH5) immunogen conferred protective immunity to the host in human-compatible vaccine dose formulations (Douglas *et al*., 2015). Historically, NHPs have been used to evaluate several malaria vaccine candidates including those based on the AMA-1 RON2 complex (David *et al*., 1985; Deans *et al*., 1988; Srinivasan *et al*., 2017) and chemically attenuated blood-stage parasites (De *et al*., 2016). NHPs (*rhesus* monkeys) were also used (for the first time) to demonstrate parasite antigenic plasticity to monoclonal antibody inhibition in some of the early malaria target-based immunization strategies. Immune sera to *P. knowlesi* PK140 resulted in rapid evolution and replacement of antibody binding epitopes with mutant forms (David *et al*., 1985). Even though some of these hurdles can or have been overcome (recently) by improved structural resolution of vaccine targets and better vaccine designs (Duffy and Patrick Gorres, 2020), the potential for these perfectly plastic parasites that can rapidly escape neutralizing effects of vaccines was first demonstrated using NHPs and are at present important considerations in malaria vaccine designs.

### Efficacy models of antimalarial drug action and resistance

In antimalarial drug discovery programmes, animal models of malaria are widely used to evaluate *in vivo* efficacy and physiological context of the drugs before human phase clinical trials (Fidock *et al*., 2004). These pre-clinical evaluations allow for deconvolution of *in vivo* drug potency, pharmacodynamics/pharmacokinetics (absorption, metabolism, distribution and excretion; ADME) and toxicities. Such profiles fine tune the discovery pipeline by selecting antimalarial agents with better potency and safety landscapes in a hit to lead optimization strategies. Animals have also been used to decipher the genetic basis of antimalarial drug resistance through *in vivo* drug selection and more recently to validate some *in vivo* phenotypes of orthologous drug resistance markers identified in *P. falciparum*.

### Hit to lead optimization of antimalarial drug candidates

**Fig. 3.**
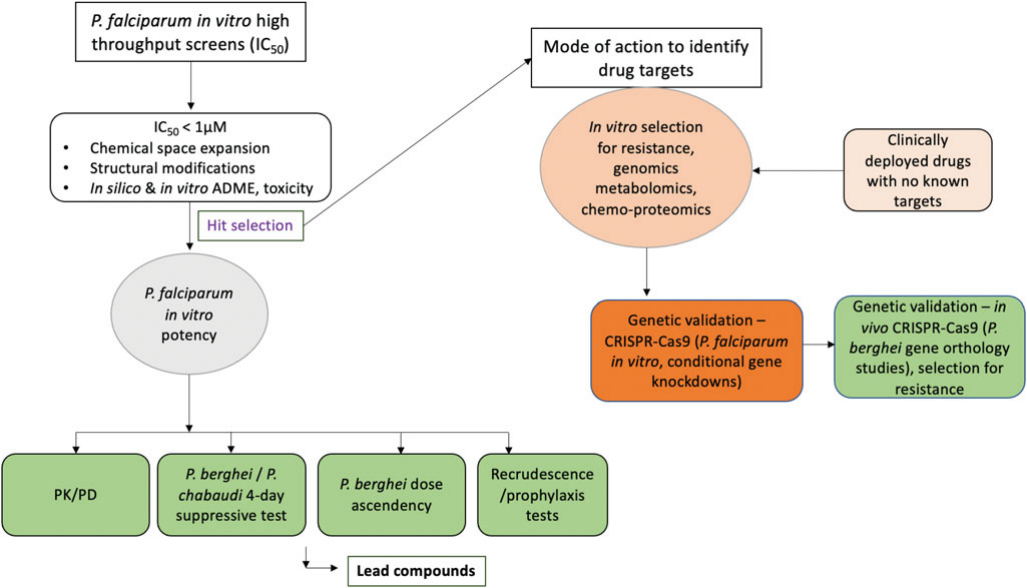
A simplified flow chart of the antimalarial drug discovery pipeline showing the utility of animal models. Discovery programmes usually start with *in vitro* whole cell phenotypic screens. Compounds to be progressed are selected based on the established half-inhibitory concentrations (IC_50_) cut-off values. These benchmarks vary between different programmes but usually compounds which achieve a <1 *μ*m IC_50_ are preferred. Selected hits are either chemically refined, expanded and or profiled for pharmacodynamics (PD) and pharmacokinetics (PK) profiles *in silico* and *in situ*. Refined hits are re-evaluated for their potency *in vitro* against *P. falciparum* and later *in vivo* in selected animal models. *In vivo* evaluations may include PD/PK analysis, suppressive test, dose ascendency and recrudescence assays. Based on established benchmarks, lead compounds are progressed to human trials. During hit selection, several approaches may be used to identify the compound molecular and biochemical targets. These may include selection for resistance and forward genetic screening, proteomics and metabolomics. Target pathways are then validated using reverse genetics approaches by CRISPR-Cas9 or other gene knockout/knockdown approaches. In certain instances, compounds can be progressed without knowledge of the drug target. In these cases resistance can be identified in field isolates and the mutations retrospectively mapped to identify the drug targets and mode of resistance. Stages where animal models are often used are highlighted in light green.

Antimalarial drug discovery programmes are mostly based on *in vitro* whole cell phenotypic screens of *Plasmodium* parasites within host red cells (Guiguemde *et al*., 2010; Chatterjee and Yeung, 2012) even though target-based screens (utilizing recombinant *P. falciparum* proteins) have become an option recently (Phillips *et al*., 2015; Alam *et al*., 2019). Phenotypic screens yield hundred to thousands of chemical scaffolds which are prioritized based on *in vitro* antimalarial activity and *in silico*-predicted physicochemical properties (ADME). Scaffolds with better profiles in the above assays are further refined to achieve better antimalarial potency or improve their ADME and toxicity safety scores. New scaffolds which pass the established benchmarks are then tested for *in vivo* efficacy and safety, usually in a small animal model. These *in vivo* proofs of concept studies often utilize RMPs, particularly *P. berghei* and *P. yoelii* (Fidock *et al*., 2004). *P. berghei* remains the most commonly used RMP for this purpose (Fidock *et al*., 2004). However, the choice of rodent parasite spp. can also depend on the antimalarial drug candidates under evaluation or the drug targets under investigation. For instance, pyridones, compounds targeting the cytochrome *bc1* complex, have been evaluated in *P. yoelii* because of the close genetic similarity of this target protein between *P. yoelii* and *P. falciparum* as opposed to the slightly divergent *P. berghei* (Yeates *et al*., 2008). Moreover, *P. berghei* is inherently less susceptible to some classes of endoperoxides (Lee *et al*., 2018; Simwela *et al*., 2020*a*, 2020*b*) while *P. yoelii* is naturally resistant to quinolines (Warhurst and Killick-Kendrick, 1967). Some of the differences in drug susceptibility between RMPs (*P. berghei*, *P. yoelii*) and *P. falciparum* could be due to reticulocyte preferences for the former which provide rich metabolic energy sources with the potential to cushion the killing effect of antimalarial agents (Srivastava *et al*., 2015). Care should, therefore, be taken when using these models for evaluating different classes of antimalarial agents, particularly metabolism-based inhibitors. The most widely used initial screen in these RMPs is the Peter's 4 day suppressive test (Peters and Robinson, 1999*a*). In this test, mice are infected with fixed parasite inocula and dosed with the drug (through appropriate channels) a few hours post-infection for four consecutive days. Parasitaemia in treated mice is then compared to untreated controls on day four post infection from which percentage suppression is usually calculated in further reference to a known antimalarial agent with proven and quantified activity. Compound series which achieve superior suppression in the four-day suppressive test are then further tested in follow up assays such as dose-escalation studies to determine the 50 and 90% effective (i.e. parasite death) doses (ED50, ED90). Further assessments of *in vivo* drug potency, bioavailability, toxicity and mode of delivery are also carried out (Fidock *et al*., 2004). Other tests may include recrudescence assays where candidate antimalarial agents are evaluated on their ability to suppress parasites for a specific duration of time post-treatment and prophylaxis potential where compounds are administered first before infection and their preventive potential assessed in daily follow-ups. Compound series that perform best in the above benchmarks are either expanded for further lead optimization or progressed through candidate selection filters before embarking on clinical development programmes. In either case, RMPs are used in evaluating further leads, re-optimizing expanded leads and or evaluating their overall potency (Fig. 3). RMPs can also be used to assess *in vitro* potency of antimalarial agents in short-term maturation assays (Franke-Fayard *et al*., 2008). This can provide necessary bridging information to explain potential discrepancies between *in vitro* (*P. falciparum*) and *in vivo* (*P. berghei*) sensitivities for some inhibitors and or whether such discrepancies are due to intrinsic differences between the parasite species or due to the pharmacodynamic/pharmacokinetic properties of the drug in the animal model. Since proof of concept in an animal model is usually required before any drug discovery programme is progressed, RMPs have, indeed, been at the front of these evaluations as most antimalarial drug candidates in clinical development at the moment (KAF156, KAE609, OZ349, DDD107498) were all shown to cure mice infected with *P. berghei* (Rottmann *et al*., 2010; Charman *et al*., 2011; Kuhen *et al*., 2014; Baragaña *et al*., 2015). Recently, drug discovery programmes are also incorporating humanized mice infected with human *P. falciparum* in evaluating the *in vivo* efficacy of antimalarial drug candidates in addition to the traditional RMPs (Coslédan *et al*., 2008; Booker *et al*., 2010; Barker *et al*., 2011; Nilsen *et al*., 2013; Baragaña *et al*., 2015). NHPs have also been used to evaluate the *in vivo* efficacy of antimalarial drug candidates, but they are at present mostly restricted to the evaluation of agents that are required for radical cure of chronic infections caused by *P. vivax* using its surrogate model, *P. cynomolgi* (Zeeman and Kocken, 2017).

### Mode of action studies and genetic basis of drug resistance

The utility of RMPs in understanding the genetic basis of drug resistance has also been exploited (Carlton *et al*., 2001). Not only have murine malaria parasites been used to confirm some of the drug resistance mutations observed in *P. falciparum* (Fig. 3), some resistance mutation alleles first identified in *P. falciparum* are now routinely used as selection markers for transfection experiments across the parasite spp. (van Dijk *et al*., 1995; Carlton *et al*., 1998*b*, 2001). Another advantage of RMPs is often the ease with which drug resistance can be selected. In *P. falciparum* forward genetics approaches, selection for drug resistance can be a long and tedious process which can take from a few weeks to years. For example, *in vitro* selection for resistance to artemisinin in *P. falciparum* took almost 4–5 years to obtain stable resistant parasites (Witkowski *et al*., 2010; Demas *et al*., 2018). On the contrary, drug resistance in RMPs, *P. berghei* and *P. chabaudi* can be selected within a short period of time. Resistance to GNF179, a related compound to KAF156 was selected after just 2 single *in vivo* dose treatments in *P. berghei* (Lim *et al*., 2016), something which took up to 4 months in *P. falciparum* (Kuhen *et al*., 2014). In *P. chabaudi*, resistance to pyrimethamine was obtained within 2 weeks after a single dose treatment (Carter and Walliker, 1975). From these pyrimethamine resistant *P. chabaudi* lines, additional lines resistant to ascending doses of chloroquine, mefloquine, artemisinin and artesunate have been easily derived (Rosario, 1976; Padua, 1981; Carlton *et al*., 1998*a*; Cravo *et al*., 2003; Afonso *et al*., 2006). Unlike in *P. falciparum*, drug resistance in rodent malarias can also be tested for *in vivo* phenotype stability in the absence of drug pressure through blood passage, freeze–thaw cycles and the mosquito infectivity and transmission filters (Rosario, 1976; Afonso *et al*., 2006). After obtaining drug-resistant parasites, genetic markers responsible for these phenotypes have been characterized by carrying out genetic crosses between sensitive parasites and resistant clones. This typically involves the transmission of sensitive and resistant parasites in a mixture into a mosquito then into a new host which allows for the selection of recombinant progenies from which chromosomal linkage analysis can be used to map candidate genes to the observed phenotypes. Even though this is also possible in *P. falciparum* (which may require NHPs or adapted humanized mouse models), RMPs are uniquely suited for such endeavours due to the ease of handling rodents and the ability to reproduce the entire *in vivo* life cycle under lab conditions (Carlton *et al*., 2001). In the meantime, the advent of recent genome sequencing technologies means candidate drug-resistant mutations in these rodent models can be quickly identified and characterized (Hunt *et al*., 2007, 2010*b*; Borges *et al*., 2011; Kinga Modrzynska *et al*., 2012). A brief role of RMPs in understanding the mode of action and resistance for principle antimalarial drugs that are and have been in clinical usage is described in detail in the following sections.

### Sulphadoxine and pyrimethamine

Resistance to antifolates is perhaps one of the well-studied and characterized resistance mechanisms in malaria parasites. Parasite resistance to antifolates is thought to occur by an initial amplification of the target locus followed by more rapid mutations that the multigenic nature of the locus facilitates which with continued selection pressure fixates into a single locus through maintenance and propagation of favourable mutations in the population (Tanaka *et al*., 1990). This also appears to be a common mechanism to early drug resistance in most eukaryotic pathogens where transport and drug efflux genes are amplified through copy number duplications before acquisition of target-specific mutations (Fairlamb *et al*., 2016). Meanwhile, resistance to sulphadoxine and pyrimethamine (SP) emerged immediately after this drug combination was rolled out for clinical use. The mechanistic details of resistance to these two drugs have now been well described (Plowe *et al*., 1997). The earliest report of an RMP resistant to pyrimethamine was in 1952 where a *P. berghei* strain with up to 20-fold resistance as compared to the wild type was obtained after two rounds of passages and treatment with single curative doses (Rollo, 1952). Several other *P. berghei* parasites resistant to pyrimethamine have also been easily obtained and reported (Diggens, 1970; van Dijk *et al*., 1994). Pyrimethamine resistance has also been selected for in the other RMPs (Yoeli *et al*., 1969; Walliker *et al*., 1973, 1975). However, the mechanism of pyrimethamine resistance in these lines was not convincingly known until mutations in the *dhfr* gene, specifically the S108N substitution, was identified in *P. falciparum* after a genetic cross of pyrimethamine-resistant field isolates with laboratory-sensitive lines (Cowman *et al*., 1988; Peterson *et al*., 1988). In these crosses, resistant parasites consistently inherited a fragment within the *dhfr* which differed from sensitive parasites by either the S108N substitutions or other candidate mutations such as N51I or C59R which are now all validated determinants of pyrimethamine resistance. Crucially, equivalent mutations in the *dhfr* such as the S106N were later identified in the *P. chabaudi* pyrimethamine resistant line (AS-Pyr) (Cheng and Saul, 1994). A similar mutation (S110N) is also responsible for the *dhfr*-mediated pyrimethamine resistance phenotype in *P. berghei* (van Dijk *et al*., 1994). Mutant *dhfr* coupled to thymidylate synthase (dhfr/ts) carrying pyrimethamine resistance alleles are currently a widely used drug selection marker for transfection experiments in both *P. berghei* and *P. falciparum* (van Dijk *et al*., 1995; Wu *et al*., 1996) as well as *P. knowlesi* (van der Wel *et al*., 1997; Mohring *et al*., 2019). Even though mutations in the *dhps* gene are predicted determinants of resistance to sulphadoxine (Plowe *et al*., 1997), this remains circumstantial due to a lack of *in vitro* assays that can reliably distinguish sulphadoxine resistant from sensitive parasites (Wang *et al*., 1997). Sulphadoxine is a sulphonamide that acts as a substrate analogue of p-aminobenzoic acid (PABA) to competitively inhibit *dhps* which in turn affects downstream folate synthesis for the parasite. Due to the variations in the levels of PABA in most culture media, phenotyping of resistance levels to sulphadoxine has been particularly difficult (Watkins *et al*., 1985; Wang *et al*., 1997). Nevertheless, transfection and allelic exchange experiments have been used to validate some of *dhps* alleles such as the A437G substitution in modulating *in vitro* susceptibility to this drug (Triglia *et al*., 1998). Due to these complexities, the genetics of dual resistance to SP drug combinations have been difficult to unravel. Parasites exhibiting SP resistance can carry mutations in the *dhfr* and *dhps* genes even though *dhfr* gene mutations alone are known to mediate resistance phenotypes to both drugs while *dhps* polymorphisms can sometimes less clearly correlate with SP resistance (Plowe *et al*., 1997). RMPs can, therefore, in these situations offer a unique opportunity for studying resistant phenotypes emanating from such drug combinations as levels of interfering parameters such as PABA can be controlled, physiologically or through artificial diet supplementation. Parasites resistant to sulphadoxine have been selected in both *P. berghei* and *P. chabaudi*, both of which appear to need less PABA as they develop an increased capacity to synthesize this metabolite *de novo* (Singh *et al*., 1954; Carlton *et al*., 2001). However, the genetic determinants in these resistant lines have remained uncharacterized. Crucially, *P. berghei* parasites resistant to SP drug combinations have been successfully obtained using a continuous low dose selection strategy even though the resistant phenotypes were unstable and the genetic determinants have not been identified (Merkli and Richle, 1983*b*). Perhaps one of the best-characterized SP-resistant RMP line is the *P. chabaudi* AS (50S/P) line. This line was obtained by a further selection of the AS-Pyr line with a single four-day high dose exposure with the SP drug combination to obtain parasite progenies that were strongly resistant to the drug combination. However, quantitative trait loci and genetic analysis of the AS (50S/P) line revealed that *dhfr* mutations were the major determinant of the SP drug resistance phenotype as no additional *dhps* mutations were identified (Hayton *et al*., 2002).

### Artemisinin

**Fig. 4.**
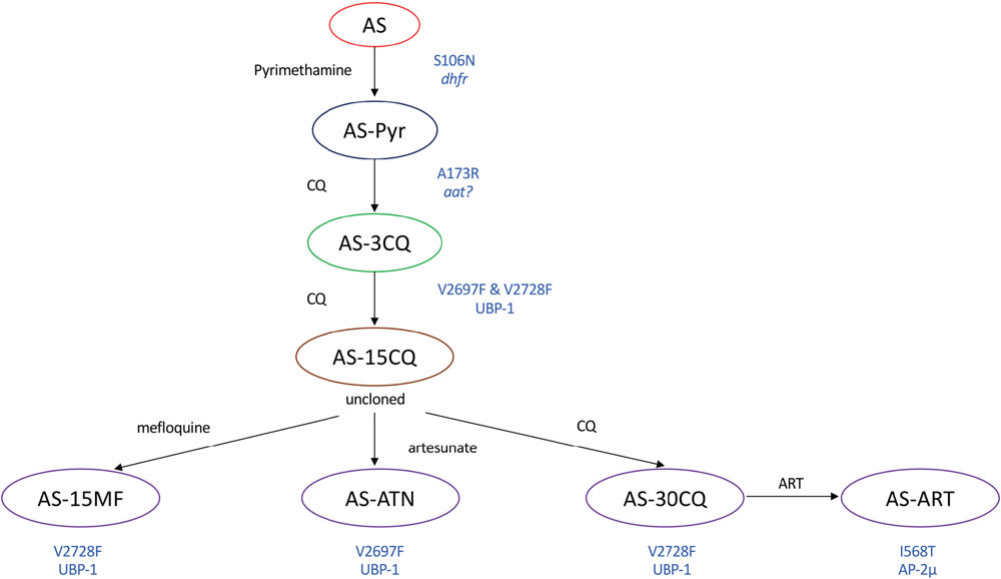
Selection strategies used to obtain *P. chabaudi* artemisinin-resistant lines and the causal genetic determinants. The original AS line was subjected to four daily doses of pyrimethamine to obtain the AS-Pyr resistant line that carry the S106N mutation in the *dhfr* gene. Further selection of this line with chloroquine (CQ) resulted in the AS-3CQ resistant line that was able to tolerate up to six consecutive doses of CQ at 3 mg kg^−1^. Whole-genome sequencing identified the A173R mutation in an amino acid transporter (*aat*) to be responsible for this phenotype. Further selection of this line with incremental doses of CQ resulted in the AS-15CQ line which carried two UBP-1 mutations, V2697F and V2728F. From this line, incremental dose selection with mefloquine, artesunate and further high doses of CQ yielded the AS-15MF, AS-ATN and AS-30CQ which appear to have fixated the UBP-1 mutations differently as indicated. Selection of the AS-30CQ line with ART resulted in the AS-ART line which carries an additional mutation in the AP-2 *μ* gene. Adapted from Hunt *et al*. (2010*b*), Henriques *et al*. (2013).

Artemisinins (ARTs) are frontline antimalarial drugs in ART-based combination therapies, their deployment of which has played significant roles in alleviating the global malaria disease burden (WHO, 2020). However, resistance to ARTs has emerged and is now endemic in most parts of SEA (WHO, 2020). ART resistance is primarily conferred by polymorphisms in a Kelch13 protein (Ariey *et al*., 2014) even though several other determinants such as UBP-1, Pfcoronin, falcipains and AP2-*μ* have been implicated (Hunt *et al*., 2007; Henriques *et al*., 2013; Demas *et al*., 2018; Rocamora *et al*., 2018). Meanwhile, RMPs have been instrumental in identifying some determinants of resistance to ARTs. ART resistant *P. berghei* and *P. yoelii* parasites were first selected and reported in the late 1990s (Peters and Robinson, 1999*a*). This involved the application of the 2% relapse technique where parasites are inoculated into mice and treated with a high subcutaneous dose of the drug 3-h post infection. Upon recrudescence, parasites are passaged into a new host and retreated with similar drug doses. Levels of resistance are quantified by graphing the changes in time required to reach 2% parasitaemia in the treatment group which can be graded as a progressive reduction in the time required to reach 2% parasitaemia over the course of the passages when resistance is successfully obtained. Using these approaches, resistance to ARTs was obtained in both *P. berghei* and *P. yoelii* which was, however, unstable as resistant parasites of both species easily lost the phenotype when the drug was withdrawn (although the lines were uncloned and overgrowth by wildtype sensitive parasites could have occurred) (Peters and Robinson, 1999*b*). Biochemical characterization of one of these ART-resistant *P. yoelii* strain which displayed up to four-fold resistance compared to the sensitive lines revealed a reduced accumulation of the radiolabelled drug in the resistant parasites (Walker *et al*., 2000). Another selection for ART resistance in *P. berghei* was attempted using the ART derivative artemether in the early 2000s (Xiao *et al*., 2004). Infected mice were treated with high doses of artemether, passaged into a new host upon recrudescence and retreated every passage for 50 passages. Even though up to 8-fold resistance was achieved in these lines, the phenotype was unstable as drug sensitivity was retained after a few rounds of infections without drug pressure. The most studied RMPs which displayed stable ART resistance phenotypes were obtained in *P. chabaudi* and were first reported in 2006 (Afonso *et al*., 2006). These lines were selected from *P. chabaudi* clones which had and were already previously selected for resistance to pyrimethamine and chloroquine (Fig. 4). The original *P. chabaudi* AS isolate was exposed to four consecutive doses of pyrimethamine at 50 mg kg^−1^ from which a pyrimethamine resistant line (AS-Pyr) was obtained and cloned (Walliker *et al*., 1975). The AS-Pyr line was then selected for resistance to chloroquine from which a line resistant to six consecutive doses of the drug at 3 mg kg^−1^ (AS-3CQ) was obtained (Rosario, 1976). Selection of the AS-3CQ line with a stepwise chloroquine dose increment yielded a *P. chabaudi* line that was resistant up to six consecutive daily doses of chloroquine at 15 mg kg^−1^ (AS-15CQ) (Padua, 1981). From the AS-15CQ line, several lines with differing drug resistance phenotypes were subsequently obtained. Exposure of the AS-15CQ line to a gradual ascending dose of mefloquine (7–30 mg kg^−1^) resulted in the parasite line that was initially resistant to up to four consecutive doses of mefloquine at 30 mg kg^−1^ but eventually lost some degree of resistance by only surviving four consecutive doses of the drug at 15 mg kg^−1^ (designated AS-15MF) (Cravo *et al*., 2003). The AS-15CQ line was also subjected to a further chloroquine selection until a line resistant to up to 30 mg kg^−1^ of chloroquine (AS-30CQ) was obtained (Padua, 1981; Carlton *et al*., 1998*a*). Selection of AS-30CQ with gradually increasing doses of ART (up to 300 mg kg^−1^) yielded an ART-resistant line (AS-ART) that survived three to five consecutive doses (up to 300 mg kg^−1^) of the drug (Afonso *et al*., 2006; Henriques *et al*., 2013). From the AS-15CQ, another selection with incremental doses of artesunate also yielded an independent line (AS-ATN) that was resistant to artesunate surviving up to 60 mg kg^−1^ of the drug (Afonso *et al*., 2006) (Fig. 4). Interestingly, both the AS-30CQ and AS-ART appear to have shared a cross resistance to the ART phenotype (Henriques *et al*., 2013). Three independent genetic crosses of the AS-ART line with a parallel *P. chabaudi* sensitive line and follow-up linkage group selection analysis identified a selection valley on chromosome 2 on the resistant parasites that strongly associated with the ART resistance phenotype. Within this locus, two mutations in a deubiquitinating enzyme, UBP-1 (close human homologue USP7) appear to have fixed independently in the AS-ATN (V2697F), AS-30CQ and AS-ART line (V2728F) (Hunt *et al*., 2007). Meanwhile, whole-genome sequencing further revealed that the V2728F mutation was not just common to the AS-30CQ and AS-ART lines, but also in the AS-15MF line (Hunt *et al*., 2010*b*). Prior analysis of the AS-Pyr already identified the expected S106N *dhfr* gene mutation that was chiefly responsible for the pyrimethamine resistance phenotype (Cheng and Saul, 1994). Further genetic analysis of these lines also revealed several other polymorphisms which may have arose as a consequence of the drug selection cascade from the original AS line. These include a mutation (A173E) in the amino acid transporter (*aat*, PCHAS_1127800) that seemingly was responsible for the chloroquine resistance phenotype in the AS-3CQ line and another mutation (T719N) in a predicted unknown transporter (PCHAS_0313700) which appears to have led to the emergence of the chloroquine resistance phenotype in the AS-15CQ line (Kinga Modrzynska *et al*., 2012). Nevertheless, it appears that the two UBP-1 mutations may have arose independently in the AS-15CQ uncloned line which resulted in their specific fixation when a drug pressure of mefloquine, artesunate or a higher dose of chloroquine was applied (Henriques *et al*., 2013). Further analysis of the ART cross-resistance phenotype between the AS-30CQ and AS-ART also revealed that selection of the former line with ART led to the successful acquisition of an additional mutation (I568T) in the AP-2 *μ* gene which was responsible for the higher level of ART resistance in the AS-ART as compared to the AS-30CQ (Henriques *et al*., 2013). However, despite the observation of some of these mutations (UBP-1 and AP-2 *μ*) in *P. falciparum* clinical isolates that have presented with potential ART resistance phenotypes (Henriques *et al*., 2014), up until recently (discussed below), their role in mediating resistance to these drugs remained an association in part due to the complexity of the selection procedure with multiple drugs as well as the absence of appropriate reverse genetics approaches.

### Atovaquone

Resistance to atovaquone (a chemical analogue of coenzyme Q) is readily achieved with the acquisition of mutations in the cytochrome bc1 complex both under laboratory conditions and in clinical field settings (Srivastava *et al*., 1997; Vaidya and Mather, 2000). This was mirrored in RMPs as resistant to atovaquone has been selected for in *P. berghei*, *P. yoelii* and *P. chabaudi* (Srivastava *et al*., 1999; Syafruddin *et al*., 1999; Afonso *et al*., 2010). The *P. chabaudi* atovaquone-resistant line was selected from the AS-3CQ (Fig. 4) line after stepwise dose escalation while resistant parasites in *P. berghei* and *P. yoelii* were selected from naïve parasite backgrounds. However, in all the three parasite species, genetic analysis revealed that the resistance phenotypes were due to mutations in the cytochrome bc1 complex even though independent reverse genetics have not been carried out to further validate their involvement. It should be noted that reverse genetics approaches could also be particularly difficult in these situations as the cytochrome bc1 complex is encoded by the mitochondrial genome.

### Mefloquine

Until recently, the mechanism of action of and resistance to mefloquine has remained relatively elusive. An aryl aminoalcohol, mefloquine was initially believed to act by inhibiting haem polymerization within the parasite digestive vacuole while increased copy numbers of the multidrug resistance transporter (PfMDR1) have been implicated as a mechanism of resistance (Price *et al*., 2004; Ecker *et al*., 2012). However, a more recent study has demonstrated that this drug can also act as a protein synthesis inhibitor in malaria parasites, specifically targeting the 80S ribosomal translational unit (Wong *et al*., 2017). Nevertheless, RMPs resistant to mefloquine have been selected and characterized. The most studied line is perhaps the AS-15MF which emerged from the AS drug selection panel of lines as described above (Fig. 4). This line was selected from a pre-existing chloroquine-resistant line (AS-15CQ) using an incremental dose of mefloquine until stable resistance at 15 mg kg^−1^ was obtained. Linkage and genetic cross analysis of this line further revealed that increased expression of the PcMDR1 through copy duplication was indeed a constant feature of all resistant progenies which inherited the resistant phenotypes (Cravo *et al*., 2003). However, further genetic analysis of the AS-15MF line also implicated the UBP-1 V2728F mutation (Hunt *et al*., 2010*b*). Mefloquine resistant *P. yoelii* parasites have also been selected for, even though the genetic determinants have not been characterized (Merkli and Richle, 1983*a*). In *P. berghei*, cloning and sequencing of the PbMDR1 gene from a line which was selected for and attained stable resistance to mefloquine also revealed 2–3-fold amplification of this gene in resistant parasites as compared to the sensitive wild type (Gervais *et al*., 1999). In these circumstances, even though the 80S ribosome might be the direct target of mefloquine, it can be relatively difficult to fully pinpoint the mode of action as UBP-1 and MDR1 which are implicated in the mode of resistance (just like with gene amplifications observed with antifolates, discussed above) could be involved with transport or efflux of the drug.

### Chloroquine

Since resistance to chloroquine emerged in *P. falciparum* in the 1970s, the mode of resistance remained poorly characterized until the early 2000s (Ecker *et al*., 2012). This is because it proved specifically difficult to select for chloroquine resistance in *P. falciparum* under laboratory culture conditions from naïve parasite strains. Identification of the chloroquine resistance transporter (PfCRT) as the principal determinant of resistance involved detailed genomic, biochemical and allele exchange experiments to identify a 13-exon gene within a 36 kb chromosomal fragment that strongly associated with chloroquine resistance phenotypes in resistant parasites (Fidock *et al*., 2000). RMPs have, however, provided an additional route to understanding chloroquine resistance as even though it has proved to be difficult to generate parasites with stable resistant phenotypes, a number of lines have been reported. The most widely studied RMP models of chloroquine resistance are from the *P. chabaudi* AS lineage as described in sections above (Fig. 4). The AS-3CQ was the first *P. chabaudi* line with a stable chloroquine resistance phenotype (Rosario, 1976). In this line, initial biochemical analysis revealed reduced accumulation of chloroquine in the resistant parasites as compared to their sensitive counterparts (Ohsawa *et al*., 1991). Subsequent quantitative genome sequence analysis of the AS-3CQ line also identified a single mutation in an amino acid transporter *aat* (PCHAS_1127800) that could be a possible determinant of this drug resistance phenotype (Kinga Modrzynska *et al*., 2012). However, reverse genetics approaches have not been carried out to further validate its contribution. Meanwhile, further selection of the AS-3CQ line generated higher degree chloroquine resistance lines (AS-15CQ and AS-30CQ), both of which carried UBP-1 mutations (V2697F and V2728F) as genetic determinants (Hunt *et al*., 2007, 2010*b*). *P. berghei* strains resistant to chloroquine have also been selected and reported which, however, has in most cases resulted in relatively unstable phenotypes. In some of the early works, exposure of *P. berghei* parasites to chloroquine supplied in animal diet for 4 months resulted in highly resistant strains that easily tolerated above maximum effective doses of the drug under standard treatment conditions (Hawking, 1966). These chloroquine-resistant parasites also displayed a cross-resistance phenotype to other drugs such as sulphadiazine and pyrimethamine. However, the phenotype was easily lost and could not be resuscitated when drug pressure was reapplied. Around the same time, other unstable chloroquine-resistant *P. berghei* RC strains that displayed up to 60-fold resistance as compared to the wild type were also reported (Peters, 1965). The only *P. berghei* parasites with stable chloroquine-resistant phenotypes were reported in 1998. These lines were selected from the NK65 line with an incremental dose of chloroquine ranging from 1, 3, 6, 10 through to 30 mg kg^−1^. From each of these doses, stable phenotypes were obtained at various levels of the drug selection (CQR3, CQR6, CQR10 and CQR30) which, crucially, displayed high-level resistance in both the *P. berghei in vitro* short-term assay as well as *in vivo* (Platel *et al*., 1998). Nevertheless, the genetic determinants of the chloroquine resistance phenotypes in these lines have not been characterized.

### Allelic exchange and Crispr-Cas9: of malaria mutant models in mice, a new approach?

RMPs (except *P. vinckei*) are easily amenable to genetic manipulation as they demonstrate higher transfection efficiency as compared to *P. falciparum* (Janse *et al*., 2006; Jongco *et al*., 2006). Reverse genetics approaches by introducing *P. falciparum* drug resistance alleles in rodent parasites through allelic exchanges have been pursued in order to assess drug resistance phenotypes *in vivo* (Fidock *et al*., 2004). PfCRT mutant forms responsible for chloroquine resistance have been introduced in *P. berghei* (Ecker *et al*., 2011). Even though these PfCRT alleles did not result in equivalent chloroquine resistance phenotypes as observed in *P. falciparum*, it was demonstrated that under drug pressure, *P. berghei* parasites carrying PfCRT mutant forms achieved better and efficient transmission. An inherent disadvantage of such approaches is that it involves the introduction of a transgene into parasites that maintain normal expression of the internal loci irrespective of how conserved the alleles are between the spp. In such situations, it is difficult to quantify drug resistance phenotypes especially if the markers under study occur in proteins or enzymes that do not respond to (trans)gene dosage or if the background expression is sufficient to overshadow any resulting phenotype from the introduced alleles. However, with the development of recent highly precise genome editing technologies such as CRISPR-Cas9, instead of introducing *P. falciparum* antimalarial drug-resistant candidate alleles in RMPs as transgenes, orthologous polymorphisms can be introduced with precision in attempts to characterize phenotypes based on assumed gene function conservation. Such CRISPR approaches have recently been used in *P. berghei in vivo* to validate UBP-1 and Kelch13 ART resistance mutations which were first identified in *P. chabaudi* and *P. falciparum*, respectively (Simwela *et al*., 2020*a*, 2020*b*). Even though evolutionary divergence between RMPs and *P. falciparum* means overall protein sequence identities cannot be always highly conserved (e.g. *P. berghei* and *P. falciparum* UBP-1 share 41% sequence identity), there appears to be a reasonable degree of similarity in the functional domains (Simwela *et al*., 2020*a*). Meanwhile, certain drug resistance genes are highly conserved, for instance *P. berghei* and *P. falciparum* Kelch13 share over 80% sequence identity (Simwela *et al*., 2020*b*). In either cases, these features have been exploited in CRISPR-Cas9 mediated reverse genetics approaches to confirm the causality of drug resistance mutant alleles in *in vivo* conditions (Simwela *et al*., 2020*a*, 2020*b*) which has at times been problematic or debatable using *in vitro* systems in *P. falciparum* (Sá *et al*., 2018). The introduced Kelch13 and UBP-1 mutations in *P. berghei* (F448I, Y505H, M488I, R551T for the former and V2721F, V2728F for the latter) all resulted in ART or chloroquine resistance phenotypes just like their *P. falciparum* and *P. chabaudi* equivalents which were reversible when some of these mutations were reversed or repaired (Simwela *et al*., 2020*a*, 2020*b*). Moreover, mutant *P. berghei* parasites carrying drug resistance alleles (UBP-1 and some Kelch13) possessed fitness defects which are easily assessed and quantified in RMPs under physiologically relevant *in vivo* conditions. In SEA, Kelch13 mediated ART resistance is primarily associated with the C580Y mutation (Hamilton *et al*., 2019; van der Pluijm *et al*., 2019). Intriguingly, *P. berghei* equivalent of the C580Y (C592Y) mutation and the other important Kelch13 mutation, R539T (R543T) could not be introduced in this RMP in several attempts despite a high degree of conservation of the predicted Kelch13 structures and mutation locale between the two parasites (Simwela *et al*., 2020*b*). The level of Kelch3-mediated ART resistance (notably with the C580Y substitution) and the resulting fitness thereof is also dependent on the parasite genetic backgrounds (Straimer *et al*., 2015) which donate compensatory architectures to cushion the deleterious effects of the mutations (Miotto *et al*., 2015). In these cases, CRISPR-Cas9 genome editing in RMP as applied in *P. berghei* can provide useful information on the evolution of drug resistance in malaria parasites by revealing the impacts of specific mutations on naive parasite backgrounds. Experimental tractability of RMPs could also mean such mutant parasites could be assessed for transmission fitness in the absence and or presence of drug pressure. These mutant RMPs are also offering opportunities to assess the *ex vivo* and *in vivo* efficacy of antimalarial agents which can be used as combinational partners with ARTs to offset resistance (Simwela *et al*., 2020*b*, 2021).

## Conclusion and future directions

Laboratory malaria research has always relied on animal models which have served as *in vivo* surrogates of human infection. From basic biology study of parasites to disease pathogenesis all the way to drug and vaccine development, these models have provided crucial insights that are currently deployed at the forefront of interventional strategies used in malaria control programmes. Historically, RMPs have been widely used since their discovery in the late 1940s, in part due to their ease of use and experimental accessibility. Since the mid-1990s, RMPs have been genetically tractable with high efficiencies and provide suitable models for high-throughput genetic screens which are otherwise mostly unattainable in human *Plasmodium* spp. However, host divergences between rodents and humans as well intra-species divergence between different *Plasmodium* species always precludes the direct extrapolation of data emerging from such traditional animal models. The availability of robust experimental systems in animal models though means their utility in studying otherwise inaccessible components of human *Plasmodium* biology will remain a go to approach for the foreseeable future. For instance, high throughput genome-wide screening resources (*Plasmo*GEM) in *P. berghei* have facilitated the identification of critical drug and vaccine targetable pathways throughout the *Plasmodium* life cycle, a wealth of information which is difficult to generate for HIP (Gomes *et al*., 2015; Bushell *et al*., 2017; Stanway *et al*., 2019) although not entirely impossible (Zhang *et al*., 2018). Such high throughput, data-rich approaches also mean a reduction in the overall number of animals used in malaria research and contribute significantly to the principles of reduction, replacement, and refinement in animal usage in biomedical research. The advent and successful adaptation of CRISPR-Cas9 to malaria parasites (Lee *et al*., 2019) is also opening new frontiers such as the interrogation of gene orthology studies in the evolution of antimalarial drug resistance between humans and RMPs (Simwela *et al*., 2020*a*, 2020*b*). This remarkable new tool is already finding use not just in antimalarial drug discovery programmes but functional and translational genomics at large (Lee *et al*., 2019). Recently, humanized mice and controlled human infections (Minkah *et al*., 2018; Stanisic *et al*., 2018) are also emerging as critical links between rodent models and human malaria parasites. The current generation of humanized mice are indeed providing a wealth of *P. falciparum in vivo* data ranging from host immune responses to parasite infection, drug and vaccine evaluation as well as the basic biology of life cycle progression. However, these first-generation humanized mice suffer from poor permeation of engrafted cells while recipient mice retain some core features of immune cell compartments. These can be limiting to the study of certain aspects of parasite biology and the development of the next generation humanized mice with humanization in several tissue and organ compartments will hopefully refine and enrich host–pathogen interaction research outputs from these models (Minkah *et al*., 2018). Where feasible, controlled human infections provide dimensions rodent models cannot such as accessible means of evaluating drug and vaccine efficacy under supervised infection with isolated parasites or cryopreserved sporozoites. They are, however, expensive and the current lack of method standardization across research centres has been a bottle neck in making direct comparison of data outputs from such studies (Roestenberg *et al*., 2012). These caveats coupled to the unrepresentative nature of *P. falciparum* strains typically used in the controlled infections as well as the fact that most field infections are known to comprise of multiple strains is also a limiting factor and a scientific hurdle controlled human infection approaches must overcome. The recent development of liver-derived organoids has also provided an additional *ex vivo* tool set for probing the biology of *Plasmodium* liver stages in host cultures that closely mimic *in vivo* conditions, reviewed by Mellin and Boddey (2020). Liver organoids have been shown to robustly accommodate infection by both *P. falciparum* and *P. vivax* liver stages opening up exciting avenues in downstream liver stage biology, drug discovery and host–parasite interactions. Nevertheless, for meaningful and exhaustive outputs, harmonized and complimentary approaches to malaria research, which utilize *in vitro* approaches with HIPs and traditional animal models in parallel with the emergent controlled human infections is the ongoing approach that will hopefully arm the next decades of the fight against malaria.
